# Effects of theta burst peripheral magnetic stimulation on cortical excitability and upper limb function after stroke: protocol for a randomized controlled trial

**DOI:** 10.3389/fneur.2025.1702806

**Published:** 2025-12-02

**Authors:** Xiaoying Lin, Ketao Du, Rongqi Ding, Qiuyu Chen, Xiaomin Niu, Qinjie Yang, Chunyang Liao, Tiantian Xin, Shuqin Li, Xiaying Fu, Jianghua Cheng

**Affiliations:** 1 Department of Rehabilitation Medicine, South China Hospital, Medical School, Shenzhen University, Shenzhen, Guangdong Province, China; 2 Jilin Medical University, Jilin City, Jilin, China; 3 Yunnan University of Business Management, Kunming, Yunnan, China; 4 Department of Rehabilitation Medicine, Dongguan Huangjiang Hospital, Dongguan, Guangdong Province, China

**Keywords:** peripheral magnetic stimulation, theta-burst stimulation, upper limb function, spasticity, non-invasive technique

## Abstract

**Introduction:**

Post-stroke spasticity affects 25%−40% of survivors, causing pain and functional impairment. Current non-invasive treatments target either central or peripheral pathways alone. This neglects the critical imbalance between spastic agonists and antagonists. We designed this protocol to address this gap through dual-site peripheral theta-burst stimulation.

**Methods:**

This single-center, three-arm, sham-controlled, single-blind randomized controlled trial enrolled 54 stroke survivors (aged 40–80, 3–12 months post-stroke, Modified Ashworth Scale score ≥1). Participants were randomized in a 1:1:1 ratio to receive a 10-session intervention over 2 weeks (5 days/week). Each session consisted of stimulation followed by 30 min of conventional physical therapy. The stimulation protocols were: Group 1: active iTBS over extensor carpi radialis longus + sham cTBS over flexor carpi radialis; Group 2: sham iTBS + active cTBS; Group 3: active iTBS + active cTBS. Stimulation was delivered using a MagTD stimulator at 110%−120% of the peripheral motor threshold (mean 38.4% maximum stimulator output), with 600 pulses per session (50 Hz burst frequency, 5 Hz theta rhythm).

**Anticipated results:**

We hypothesize that Group 3 (combined stimulation) will yield the greatest reduction in spasticity (MAS decrease ≥ 1) and the most significant improvements in upper-limb function (FMA-UE, ARAT) compared to Groups 1 and 2. These clinical gains are expected to correlate with neurophysiological changes (MEP amplitude, cortical silent period), supporting the central-peripheral synergy model.

**Discussion:**

This protocol tests a novel dual-site pTBS paradigm. Positive findings would provide preliminary evidence for network-based rehabilitation. This could inform larger trials and potentially transform post-stroke spasticity management.

## Introduction

Spasticity, a common yet not obligatory feature of upper motor neuron syndrome, often accompanies other sensorimotor deficits after central nervous system injury (1). Epidemiological data indicate that post-stroke spasticity occurred in 25.3% of all survivors, 26.7% of first-ever cases, and 39.5% when paresis was present. Among paretic patients, 9.4% had disabling/severe spasticity (Modified Ashworth Scale, MAS > 3) and 10.3% had severe spasticity (2). Clinically, spasticity is a key contributor to pain, joint contractures, impaired volitional movement, caregiver burden, and diminished quality of life (3). Although they are widely used, early rehabilitation, oral antispastic drugs, botulinum-toxin injections, and surgical decompression often confer only transient benefits and carry risks of systemic adverse effects or invasive complications (4–7). This underscores the critical demand for a non-invasive, precisely targeted, and sustainable neuromodulatory strategy to address post-stroke spasticity.

In recent years, central neuromodulation techniques—most notably repetitive transcranial magnetic stimulation (rTMS)—have shown promise in modulating cortical excitability and promoting neuroplasticity (8). Among these, theta burst stimulation (TBS) has garnered particular attention due to its time efficiency and polarity-specific effects:intermittent TBS (iTBS) facilitates cortical excitability, whereas continuous TBS (cTBS) exerts inhibitory effects (9). However, the majority of existing TBS studies have focused exclusively on the contralesional primary motor cortex (M1), with limited direct impact on peripheral spastic musculature. Although some studies have reported that central TBS can improve global upper-limb function, systematic reviews have noted that significant reductions in spasticity have not been consistently observed, with inconsistencies largely attributable to heterogeneity in stimulation parameters across studies (10). Therefore, current evidence suggests that targeting a single central site may be insufficient to address the multifactorial pathophysiology underlying post-stroke spasticity, which involves maladaptive remodeling across the cortex-spinal cord-muscle axis.

Repetitive peripheral magnetic stimulation (rPMS) delivers pulsed magnetic fields to peripheral nerves and muscles and is increasingly used for post-stroke upper-limb rehabilitation. Among various rPMS protocols, the theta-burst stimulation pattern—hereafter referred to as peripheral theta-burst stimulation (pTBS)—offers short-duration, polarity-specific plasticity induction. Among rPMS patterns, pTBS (including intermittent iTBS and continuous cTBS bursts) offers short-duration, polarity-specific plasticity induction. Hereafter we use pTBS to denote the rPMS protocol employed in this trial. By inducing afferent volleys and modulating synaptic plasticity at both spinal and cortical levels, pTBS may facilitate sensorimotor recovery (11). A recent systematic review indicated that pTBS exerts positive effects on muscle strength, spasticity, and functional impairment in stroke survivors (3). Nonetheless, existing studies have primarily targeted spastic muscles in isolation, yielding inconsistent outcomes in terms of muscle tone, passive range of motion, and voluntary control. These inconsistencies are largely attributable to heterogeneity in stimulation parameters (e.g., frequency, intensity, duration) and methodological differences in outcome assessment (12). More importantly, these investigations have largely overlooked the synergistic imbalance between spastic agonists and their antagonists and have not systematically explored a bidirectional central-peripheral network modulation strategy.

In recent years, our understanding of neuromodulatory mechanisms has continued to evolve. The “W-shaped” nonlinear model, originating from research on cognitive aging, posits that the relationship between cortical excitability and neural function is not linear (13). This model asserts that both excessively high and low states of cortical excitability are maladaptive, leading to impaired behavioral performance, while optimal cognitive function corresponds to an intermediate “optimal functional zone.” Although this model was developed in the cognitive domain, the core principle it reveals—the “excitability-function relationship” —likely represents a universal operating principle for neural networks. This is corroborated by the work of Buss et al., (14) which demonstrated that in cognitively healthy older adults, higher cognitive reserve is associated with lower motor cortex excitability. This suggests that a more optimized, moderate level of excitability forms the foundation for efficient neural processing. Critically, this principle provides a novel, integrative lens through which to understand post-stroke motor dysfunction. The classic post-stroke imbalance between hemispheres—characterized by hypoexcitability in the affected motor cortex and hyperexcitability in the unaffected hemisphere—precisely mirrors the two dysfunctional states at the extremes of the “W-shaped” model. Therefore, synergistically driving motor cortical excitability toward the central “optimal functional zone,” rather than merely applying unidirectional facilitation or inhibition, should constitute a more effective regulatory strategy. However, existing pTBS protocols have yet to leverage this theoretical framework. They typically lack a bidirectional design capable of simultaneously driving both hyper- and hypo-excitable neural circuits toward the “optimal functional zone” in an agonist-antagonist manner.

Building on this framework, we designed an RCT to formally test these mechanisms and establish clinical feasibility. This protocol makes three contributions: (1) a standardized PMT titration method, (2) first RCT testing the W-shaped model through dual-site pTBS, and (3) a rigorous sham-controlled design. Primary objectives are to evaluate pTBS effects on spasticity/function, investigate neurophysiological mechanisms, and establish safety profiles. Secondary objectives include validating central-peripheral synergy, comparing single vs. dual-site stimulation, and generating effect sizes for future trials.

## Methods

### Study design

**Figure 1:**
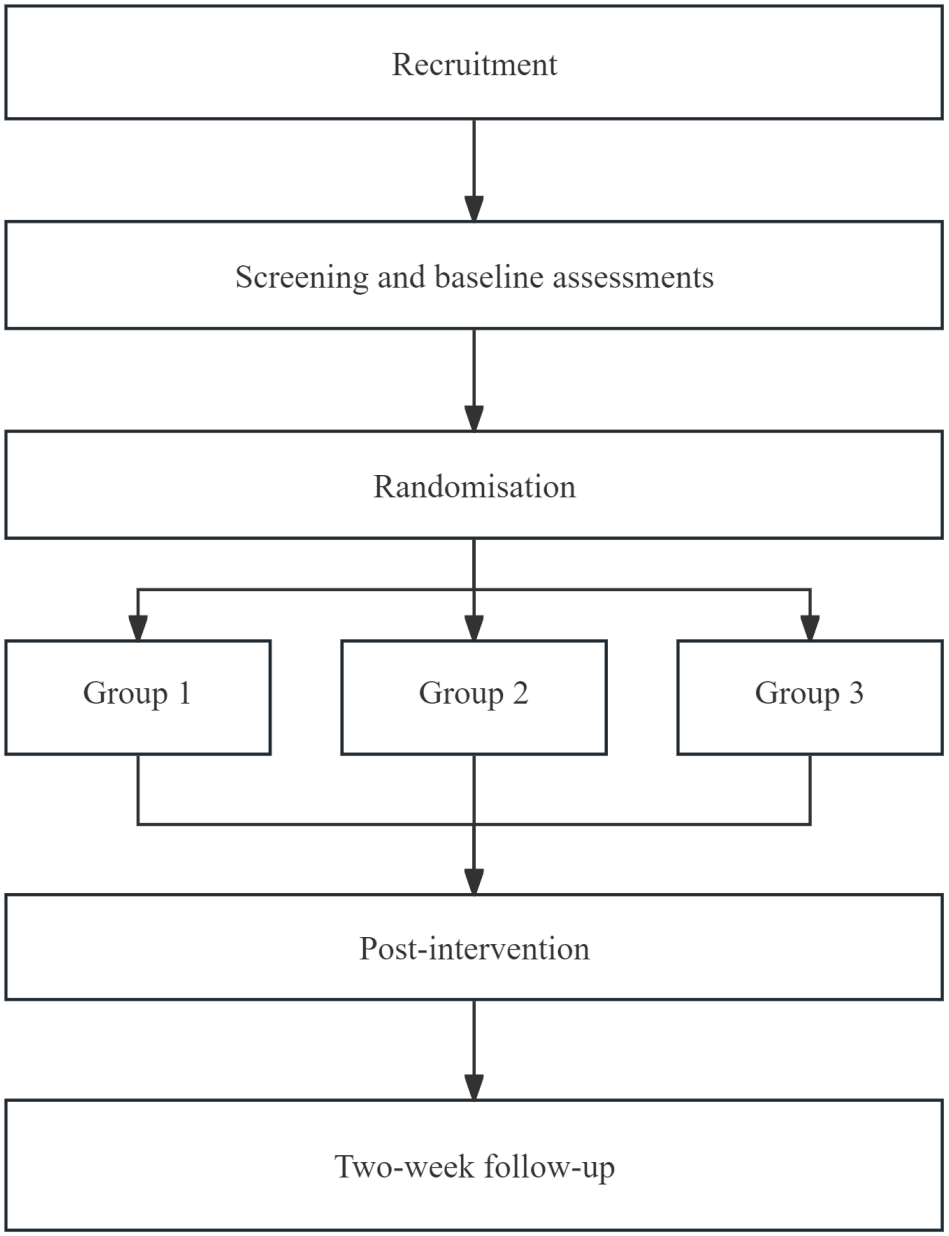
Flowchart of the proposed randomized controlled trial. Group 1: Active iTBS + sham cTBS combined with CPT; Group 2: sham iTBS + active cTBS combined with CPT; Group 3: Active iTBS + active cTBS combined with CPT. CPT, conventional physical therapy; FMA-UE, Fugl-Meyer assessment for upper extremity.

The study is a prospective, single-center, randomized, parallel-controlled design. Fifty-four patients will be randomly assigned to one of three groups (Figure 1): (1) active iTBS+sham cTBS; (2) sham iTBS+active cTBS; (3) active iTBS+active cTBS in 1:1:1 ratio. The trial comprises three phases (Table 1): baseline assessment, post-training assessment (i.e., after 10 sessions) and follow-up assessment (i.e., 2 weeks after the completion of all training sessions). Intervention will be conducted five sessions a week, with a total of 10 sessions. All participants will sign an informed consent form. Adverse reactions will be handled in accordance with the preset protocol. The whole study will be performed at the Department of Rehabilitation Medicine of South China Hospital of Shenzhen University (Shenzhen, Guangdong Province, China). The results will be published in peer-reviewed journals and reported at academic conferences. The protocol is registered with Chinese Clinical Trial Registry (Registration No. ChiCTR2500105190). The trial will be conducted in accordance with the Consolidated Standards of Reporting Trials (CONSORT) guidelines, and the study protocol has been approved by the Institutional Review Board of Ethics Committee of South China Hospital of Shenzhen University (grant no. HNL.S2025041).

**Table 1:** Schedule of participant recruitment, intervention and assessments.

**Time point**	**–T1**	**T0**	**T1**	**T2**
Recruitment	√			
Eligibility screening	√			
Informed consent	√			
Randomization		√		
**Intervention**
Group 1		√	√	√
Group 2		√	√	√
Group 3		√	√	√
**Assessment**
MEPs	√	√	√	√
MAS	√	√	√	√
FMA-UE		√	√	√
ARAT		√	√	√
Modified Barthel index		√	√	√

– T1, screening; T0, baseline; T1, post-intervention; T2, 2-week follow-up; MEPs, motor-evoked potentials; MAS, Modified Ashworth Scale; ARAT, action research arm test.

### Sample size estimation

As the differential effects of priming iTBS on hemiparetic upper-limb training have yet to be examined, we based our sample-size estimate on existing three-group trials. A recent meta-analysis reported a pooled Cohen's *d* = 0.60 favoring iTBS for upper-limb motor improvement. For a three-group design, this translates to an effect size *f* of ~0.30 (15). *A priori* power analysis (power = 0.80, two-tailed α = 0.05) indicated that 45 participants would be required. Accounting for an anticipated 15% dropout rate, we will recruit 18 participants per group, for a total sample size of 54. This recruitment target is expected to yield at least 45 participants completing the trial.

### Randomization and blinding

Randomization will be performed using a computer-generated random sequence by an independent statistician who is not involved in participant recruitment or outcome assessment.

Group allocation will be concealed in sequentially numbered, opaque, sealed envelopes, which will be opened by the intervention therapist immediately prior to the first treatment session. Both participants and outcome assessors will be blinded to group allocation. The intervention devices for active and sham stimulation will be identical in appearance and generate similar auditory and tactile sensations. For sham stimulation, the coil will be positioned at an angle that prevents effective peripheral stimulation while maintaining the same sound and vibration as active stimulation. Treating therapists delivering the stimulation will not be involved in outcome assessment or statistical analysis to maintain assessor blinding and minimize bias.

### Inclusion and exclusion criteria

We will recruit adults aged 40–80 years who sustained their first-ever unilateral ischemic or hemorrhagic stroke 3–12 months earlier, as confirmed by Computed Tomography/Magnetic Resonance Imaging and classified according to World Health Organization or International Classification of Diseases criteria. Participants must present with moderate upper-limb —impairment defined as a MAS score ≥ 1 or a Fugl-Meyer Upper Extremity score between 20 and 50 and be able to understand and follow single-step verbal instructions. All subjects must sign written informed consent. Individuals will be excluded if they have epilepsy, intracranial hypertension, or any implanted metal devices; unstable medical conditions such as severe arrhythmia, acute infection, or hepatic/renal failure; pregnancy or lactation; recent (past 3 months) craniotomy, botulinum-toxin injections, or high-dose centrally acting antispasmodics; unhealed fractures, severe arthritis, or skin lesions in the upper limbs; cognitive impairment (Mini-Mental State Examination < 24) or severe psychiatric illness/substance abuse; or previous intolerance to magnetic stimulation (Table 2).

**Table 2:** Inclusion and exclusion criteria.

**Inclusion criteria**	**Exclusion criteria**
Aged 40–80 years	History of epilepsy or intracranial hypertension
First-ever unilateral ischemic or hemorrhagic stroke, 3–12 months post-stroke	Any implanted metal devices (e.g., pacemaker, cochlear implant)
MAS score ≥ 1 in the affected upper limb	Unstable medical conditions (e.g., severe arrhythmia, acute infection, hepatic/renal failure)
FMA-UE score between 20 and 50	Pregnancy or lactation
Able to understand and follow single-step verbal instructions	Botulinum toxin injection in the affected upper limb within the past 3 months or recent craniotomy
Provides written informed consent	Unhealed fractures, severe arthritis, or skin lesions in the upper limbs
	Cognitive impairment (MMSE < 24) or severe psychiatric illness/substance abuse
	Known previous intolerance to magnetic stimulation

### Theta burst stimulation protocol targeting antagonist and spastic muscles

The flexor carpi radialis (FCR) and extensor carpi radialis longus (ECRL) were selected as stimulation targets based on their established role as a primary agonist-antagonist pair responsible for the wrist flexion-extension imbalance in post-stroke upper limb spasticity. The central mechanistic hypothesis is that the spatial proximity of their cortical representations within the primary motor cortex enables the simultaneous modulation of this pair to preferentially target a shared cortical microcircuit, thereby regulating its excitatory-inhibitory balance to reset abnormal motor output (16).

**Figure 2:**
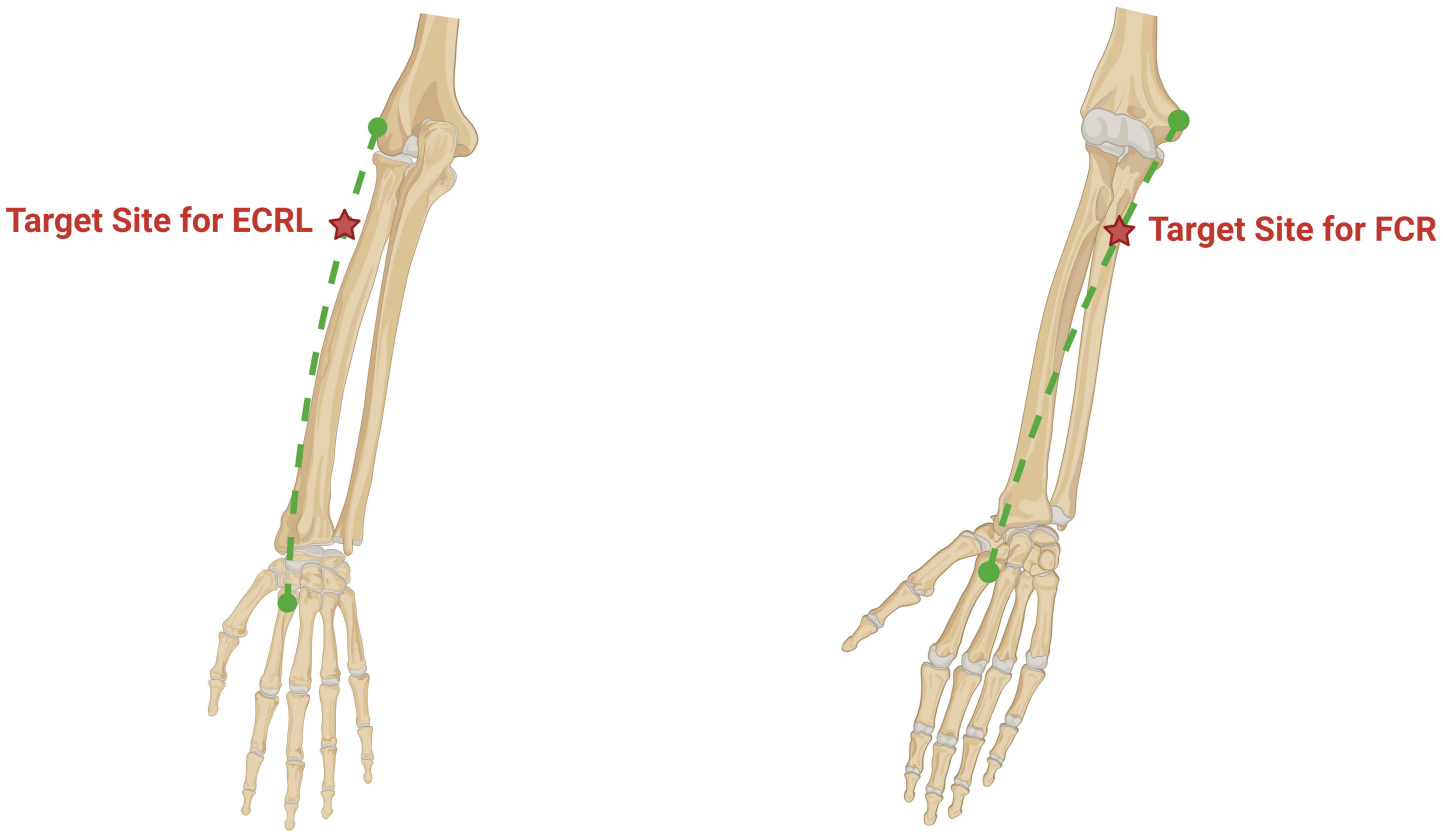
An anatomical figure for targeting landmarks. Left image: The target muscle is the extensor carpi radialis longus. Its origin is the lateral epicondyle, and its insertion is the dorsal surface of the base of the second metacarpal bone, connected by the green dashed line. The red five-pointed star indicates the stimulation point location, which is 4 cm distal to the lateral epicondyle. Right image: The target muscle is the Flexor Carpi Radialis. Its origin is the medial epicondyle, and its insertion is the palmar surface of the base of the second metacarpal bone, connected by the green dashed line. The red five-pointed star indicates the stimulation point location, which is 5 cm distal to the medial epicondyle.

**Figure 3:**
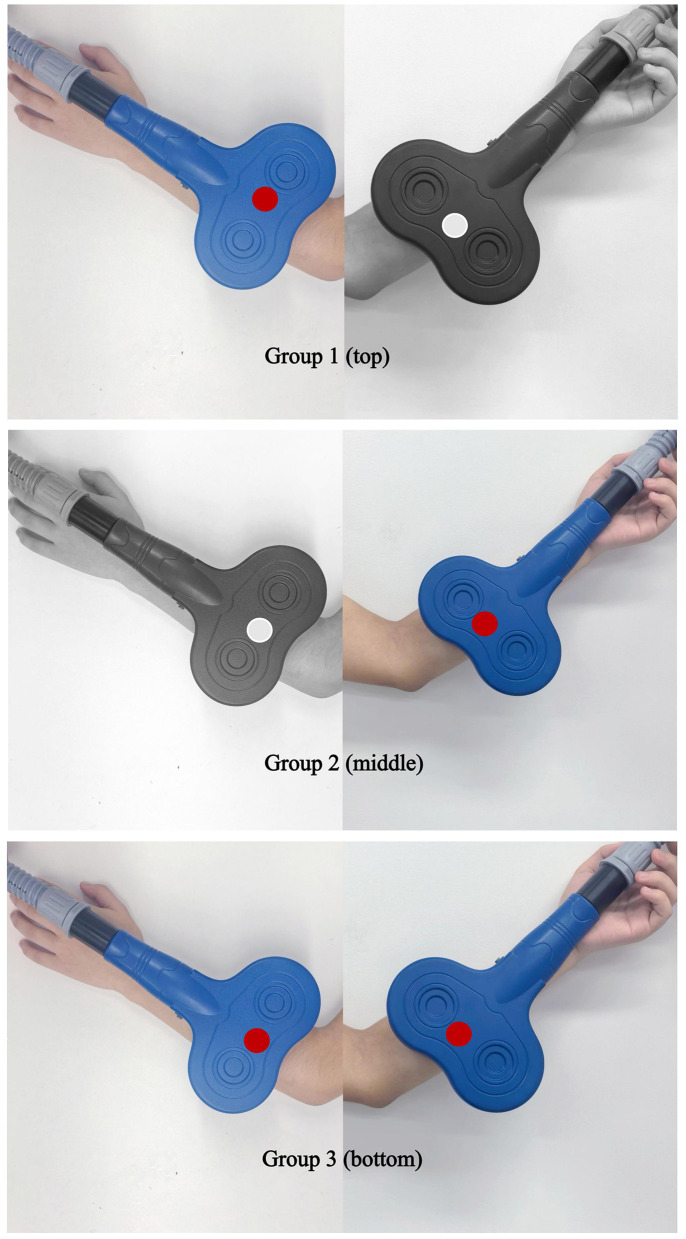
Schematic of peripheral coil placement for the three trial arms. Group 1 **(top)**: Active iTBS over ECRL (antagonist) + sham cTBS over FCR (spastic muscle). Group 2 **(middle)**: Sham iTBS over ECRL + active cTBS over FCR. Group 3 **(bottom)**: Active iTBS over ECRL + active cTBS over FCR.

All enrolled participants will receive iTBS, cTBS or sham stimulation before CPT session each weekday, Group 1: active iTBS to the antagonist plus sham cTBS to the spastic muscle. Group 2: sham iTBS to the antagonist plus active cTBS to the spastic muscle. Group 3: active iTBS to the antagonist plus active cTBS to the spastic muscle (Figure 2). The intervention will last for two consecutive weeks, with all procedures strictly adhering to the 2021 safety guidelines issued by the International Federation of Clinical Neurophysiology (17). Stimulation will be delivered with a MagTD transcranial magnetic stimulator (MagTD; Wuhan Yiruide Medical Device Technology Co., Ltd., Wuhan, China) and a figure-of-eight double coil (maximum field strength = 2 T, diameter = 9 cm). The coil center will be placed tangentially over the target muscle group, with the induced current directed downward. For the sham stimulations, we choose the Magstim's70 mm figure-of-eight double rapid2 air cooled coil (P/N 3910-00 S/NO 728) (18). The coils were identical in external appearance which ensured that the patients remained blind to the intervention (Figure 3).

Peripheral stimulation intensity was individualized based on the peripheral motor threshold (PMT). For each participant, the coil was placed over the target muscle (FCR or ECRL) and the intensity was gradually increased from 20% of maximum stimulator output (MSO). The PMT was defined as the lowest intensity that consistently evoked a visible and palpable muscle twitch without causing pain or discomfort.

The final stimulation intensity was set at 110%−120% of the individual's PMT, corresponding to an average of 38.4% ± 9.2% MSO across all participants (range: 25%−52% MSO). This approach of titrating intensity relative to PMT is a established methodology in the field (11). The selected intensity range was designed to ensure adequate afferent input by consistently eliciting a robust M-wave, a key indicator of effective peripheral activation used in previous studies (19).

To ensure stimulation consistency across sessions, the evoked M-wave amplitude was recorded at the beginning and end of each treatment session using surface EMG (1,000 Hz sampling, 50–500 Hz bandpass filter). The target M-wave amplitude for effective stimulation was set between 5 and 10 mV, a range that indicates strong, suprathreshold activation of the peripheral nerve-muscle circuit, which is considered necessary for inducing plasticity. If the amplitude deviated by >20% from the baseline value, the stimulation intensity was recalibrated to maintain consistent peripheral activation. The overall safety of applying pTBS within these parameters in stroke survivors has been supported by systematic appraisal of the literature (20).

The iTBS protocol was configured to deliver a total of 600 pulses, organized into 20 trains. Each train consisted of 10 bursts, with an 8-s inter-train interval. Within each burst, three pulses were administered at a frequency of 50 Hz, and the bursts themselves were repeated at a theta frequency of 5 Hz. The total stimulation duration for the iTBS protocol was 200 s (Table 3). During treatment, participants will lie supine. For iTBS: the figure-of-eight double coil will be centered over the antagonist muscle—specifically, the most prominent belly of the extensor carpi radialis longus (ECRL), located 4 cm distal to the lateral epicondyle of the humerus and 1 cm radially (Figure 2). This location was confirmed through anatomical palpation and is standardized to ensure consistent targeting of the ECRL muscle belly, a common motor point for effective stimulation (20, 21). The coil plane will be flush with the skin surface, the handle oriented proximally and angled aligned parallel to the long axis of the forearm. The cTBS protocol was configured to deliver a total of 600 pulses in a single, continuous train of 200 bursts without interruption. The total stimulation duration for cTBS was 40 s, during which bursts were delivered continuously at a theta frequency of 5 Hz. Each burst contained three pulses administered at a frequency of 50 Hz. For cTBS: the coil will be centered over the spastic muscle—the most prominent belly of the flexor carpi radialis (FCR), located 5 cm distal to the medial epicondyle of the humerus and 1 cm ulnarly. Similarly, this site was determined based on anatomical landmarks to reliably target the FCR belly, promoting reproducible stimulation across sessions (20, 21). The coil plane will again lie flat against the skin, with the handle oriented proximally and aligned parallel to the long axis of the forearm. The stimulation intensity for both iTBS and cTBS was set at 110%−120% of the individual's peripheral motor threshold (PMT).

**Table 3:** Detailed parameters for the peripheral theta-burst stimulation protocols.

**Parameter**	**iTBS**	**cTBS**
Total stimulation duration	200 s	40 s
Total pulses	600	600
Pulse frequency (within burst)	50 Hz	50 Hz
Pulses per burst	3	3
Burst frequency	5 Hz	5 Hz
Bursts per train	10	200
Total trains	20	1
Inter-train interval	8 s	N/A
Stimulation intensity	110%−120% of PMT	110%−120% of PMT

iTBS, intermittent theta-burst stimulation; cTBS, continuous theta-burst stimulation; PMT, peripheral motor threshold; N/A, not applicable.

### CPT

After receiving iTBS, cTBS or sham stimulation to the spastic muscle or the antagonist, all participants will undergo a 30-min session of CPT delivered by licensed therapists. Each session will consist of physical modalities, occupational therapy task training, and manual massage/manipulation. The therapy will be provided Monday through Friday throughout the 2-week intervention period.

### Outcome measures

MEPs are compound muscle action potentials recorded from a target muscle after a single-pulse TMS over the primary motor cortex; they provide an objective index of corticospinal excitability and conduction integrity, with excellent test-retest reliability [Intraclass Correlation Coefficient (ICC) = 0.75–0.94, Kendall's tau = 0.81] (17). In this trial, MEPs will be used to quantify corticospinal plasticity before and after the intervention.

Although the pTBS intervention targets the forearm muscles (FCR and ECRL), MEPs were recorded from the abductor pollicis brevis (APB) because it is a standard target muscle in TMS studies, recommended for its consistent and robust MEP responses and well-defined cortical representation in the primary motor cortex (M1) hand area (17). This approach provides a reliable and comparable measure of overall corticospinal excitability.

Briefly, Ag/AgCl surface electrodes will be placed over the abductor pollicis brevis of the paretic limb. A Magstim 200^2^ stimulator with a 70-mm figure-of-eight coil will deliver 0.2-Hz stimuli to the affected-hemisphere M1 hand area while the participant rests. The following metrics will be obtained: (1) resting motor threshold (RMT), defined as the lowest stimulus intensity eliciting ≥ 50 μV MEPs in ≥ 5 of 10 consecutive trials (17); (2) MEP peak-to-peak amplitude (mV) measured at 120%RMT, averaged across 10 artifact-free trials, as a marker of corticospinal excitability; and (3) MEP latency (ms) from stimulus artifact to onset of the first deflection, reflecting central conduction time. All measurements will be performed by the same assessor at baseline, immediately post-intervention, and at the 2-week follow-up to ensure consistency.

The MAS is the gold-standard instrument for quantifying spasticity. For the upper limb it demonstrates good reliability: inter-rater ICC = 0.781 (95% CI 0.679–0.853), κ = 0.625 (95% CI 0.350–0.801); intra-rater ICC = 0.748 (95% CI 0.671–0.809), κ = 0.593 (95% CI 0.467–0.696) (17). Scores range from 0 (normal tone) to 4 (severe spasticity preventing joint movement), with an additional 1+ grade for mild spasticity. In this study, the same blinded assessor will evaluate the spastic FCR at baseline, immediately after the last session, and at the 2-week follow-up: the elbow is flexed 90 °, the forearm is in neutral, and the wrist is passively extended at ≈1 rad/s; a sustained decrease of ≥1 point is considered a clinically meaningful improvement.

The FMA-UE is a specialized scale designed to evaluate upper limb motor function in stroke patients. The estimated range of its clinically important difference is 4.25–7.25 points (22). It consists of 33 items, covering movements of both the proximal and distal parts of the upper limb. The total score ranges from 0 to 66 points, with each item scored from 0 points (inability to complete the action, accompanied by severe abnormal movements) to 2 points (the action is performed in a standardized way and can be completed smoothly).

The ARAT is a standardized scale used to assess the upper limb motor function of patients with neurological impairments. It has high reliability and internal validity, with an intra-class correlation coefficient ranging from 0.992 to 0.998 (23). The scale consists of 19 assessment items, with a total score of 57 points. The score for each item ranges from 0 points (poor upper limb function, unable to complete the action) to 3 points (good upper limb function, the action is standard and completed smoothly).

The Modified Barthel Index is a widely used standardized scale in clinical practice for assessing activities of daily living. It evaluates an individual's basic daily living abilities, covering 10 items such as feeding, bathing, grooming, dressing, bowel and bladder control, toilet use, transfers, ambulation or wheelchair mobility, and stair climbing. It has good reliability and internal validity, with an intraclass correlation coefficient of 0.95 (24, 25). The scale consists of 10 assessment items, with a total score ranging from 0 (completely dependent on others) to 100 (completely independent), effectively reflecting an individual's degree of self-care in daily life.

### Safety measurements

Safety monitoring: Immediately after each stimulation session, all possible stimulation-related adverse events—including headache, nausea, neck pain, seizure, mood changes, fatigue, tinnitus, dizziness, somnolence, and syncope, as well as local adverse effects such as skin redness, irritation, paresthesia, or muscle soreness at the stimulation site—will be documented on the Adverse Reaction Record Form; this approach is consistent with standard adverse-event surveillance in non-invasive brain stimulation trials (17). Any adverse events potentially linked to CPT will also be captured on the dedicated Adverse Event Case Report Form (26). The intervention will be discontinued if a participant experiences persistent, moderate-to-severe discomfort at the stimulation site or any other adverse event deemed unacceptable by the investigator.

### Statistical analysis

For this study, MEP data will be processed and analyzed statistically using the Statistical Package for the Social Sciences (SPSS, version 26.0). For normally distributed continuous data, repeated-measures analysis of variance will be used for intra-group comparisons and independent-samples *t*-test for inter-group comparisons; for non-normal data, Friedman test and Wilcoxon rank-sum test will be adopted; for count data, Pearson χ^2^ test will be employed. The intraclass correlation coefficient (ICC) will be used to assess the inter-rater reliability of MEPs and MAS (17), and Cronbach's α coefficient will be used to test the internal consistency of scales. A *p* value < 0.05 will be considered statistically significant. The measurement of MEPs will follow the guidelines of the International Federation of Clinical Neurophysiology (27), and the FMA-UE will refer to the criteria for clinically important differences (22). To investigate the therapeutic mechanism, we will examine the association between changes in neurophysiological measures and improvements in clinical behavioral outcomes. Specifically, we will calculate the change scores (from baseline to post-intervention) for each participant in MEP amplitude (or cortical silent period) and in the Fugl-Meyer Assessment for Upper Extremity (FMA-UE) scores. We will then use Spearman correlation analysis to determine whether a significant correlation exists between these two change scores. We hypothesize that participants with greater clinical improvement will also exhibit more pronounced beneficial changes in corticospinal excitability.

## Discussion

The W-shaped excitability framework—where both hyper- and hypo-excitability impair function—doesn't map neatly onto post-stroke spasticity (28). Stroke does not produce a uniform excitability shift; it may create a spatially dissociated pathology characterized by spinal hyperexcitability in spastic flexors and, as suggested by our pilot data and others, cortical hypo-excitability in their antagonists; accordingly, this “split-modulation” approach emerged from pilot data (*n* = 12) where cTBS alone reduced MAS scores without improving ARAT, and iTBS alone did the opposite—suggesting that resetting the network requires both levers (28).

Parameter selection bypassed convention in favor of peripheral nerve physiology. The 110%−120% PMT intensity—averaging 38.4% MSO in our pilot—produced a 15%−20% increase in M-wave amplitude, the narrow band where Ia fibers activate without recruiting nociceptive afferents (20). Below this threshold, spinal plasticity markers (H-reflex suppression) vanished on TMS-EEG (27); above it, tolerance dropped below 70% within 15 min. The 600-pulse dose was selected based on a balance between efficacy and safety. The IFCN guidelines recommend a maximum of 800 pulses per session for peripheral TBS protocols to ensure safety (17). Furthermore, doses of 400–800 pulses have been commonly employed in prior rPMS studies and are considered necessary to induce consistent neurophysiological changes at the spinal level, such as H-reflex suppression (11, 21). Thus, our 600-pulse protocol operates within established safety boundaries while aligning with doses used in effective rPMS studies.

The distinctive sounds and sensations produced by the active stimulation device posed a significant risk to blinding. To counter this, we implemented a comprehensive strategy. We engineered the sham coil to replicate the sensory profile of the active device and added random timing jitter to mask its rhythmic pattern. This technical approach cut the rate of correct group identification in our pilot study from 38 to 17%. Concurrently, we administered deceptive questionnaires about non-existent sensory details, a tactic that reduced participants' confidence in their guesses to chance levels. We will formally assess unblinding at the trial's conclusion and perform sensitivity analyses to gauge its influence on outcomes. For assessors, blinding was secured by physically separating them from the intervention suite, encrypting all group assignments, and employing multiple independent raters.

A single-center study with a modest sample size (*n* = 54) may limit the generalizability of our findings. Rather than dilute inclusion criteria, we embedded a mechanistic substudy in the first 30 participants: simultaneous TMS-EEG and spinal H-reflex recording to build a Bayesian hierarchical model linking individual excitability profiles to response (27). This won't boost statistical power but provides a prior for phase II trials, preventing wasted resources on suboptimal doses.

To further explore the long-term effects, a future 6-month substudy (*n* = 20) is planned. This substudy will not merely extend observation—it will use wearable EMG to continuously track the time constant of long-term trajectory of spasticity. This would reveal whether durability requires periodic booster sessions, directly informing translational decisions (home-based vs. clinic-based devices).

Based on our pilot study (unpublished), we identified a “high neural noise” phenotype, characterized by PMT variability (CV > 15%) in 23% of participants. Rather than treating this as statistical noise, we will use it as a predictive feature: poor responders with high CV will signal the need for EMG-closed-loop titration in future protocols. This is an adaptive design element nested within the trial logic, not a *post-hoc* fix.

Positive results remain contingent on three unresolved conditions: (1) Technical—can the dual-coil apparatus be miniaturized into a wearable? (2) Physiological—does efficacy depend on residual corticospinal integrity (do absent MEPs preclude benefit)? (3) Economic—does cost-effectiveness outperform botulinum toxin plus therapy in real-world settings (3, 29)? We are collaborating with a manufacturer on a dual-channel pTBS prototype, but the design metric isn't power output, but rather minimizing the “acoustic fingerprint” that undermines blinding. This engineering constraint may prove the study's most commercially relevant byproduct.

Ultimately, this RCT tests whether the W-shaped model's “optimal zone” exists at the individual level (28). Aggregated group means are secondary; single-subject excitability shifts determine the model's survival.
